# Thyroid Grafting in Myxœdema

**Published:** 1891-12-05

**Authors:** 


### SURGERY.

### THYROID GRAFTING IN MYXŒDEMA.

The fact that several cases of undoubted myxœdema have followed, and been caused by, the removal by operation of the thyroid gland, and that idiopathic myxœdema is associated with atrophy of that gland, has opened up the question of transplantation of the thyroid glands of lower animals, in the hope that by engrafting it on the patient, the ill understood functions of the absent thyroid may be performed by deputy.

Mr. Victor Horsley, encouraged by the experiments of Von Eiselsberg, suggested that the progress of myxœdema might be arrested by transplantation (into the peritoneal cavity) of a portion of the thyroid gland of the sheep. Von Eiselsberg had shown that the transplanted thyroid was capable of becoming vascularised in its new situation, and of continuing its function, since out of nine animals from which he extirpated one lobe, having previously transplanted the other into the mesentery or subperitoneal tissue, the only one which did not exhibit symptoms characteristic of total ablation, was that animal in which the transplanted lobe had neither necrosed nor degenerated.

The proposal of Professor Horsley, which was certainly remarkable, has actually been put in practice by M. Lannelongue, who reported his case at a meeting of the Paris Biological Society. The subject was a myxœdematous cretin, fourteen years of age. About two-thirds of the left lobe of the thyroid was removed from a young adult sheep and transplanted into the subcutanous tissue of the child's chest, below the right breast, it being found impossible to insert it in the neck owing to the presence of large "myxœdematous tumours" in that region. The capsule of the transplanted organ was removed before embedding it at a depth of three centimetres from the skin. The operation was aseptic, and the wound united without fever or suppuration. Whether any appreciable result will follow remains to be seen, but M. Lannelongue believes that the grafting has succeeded. Professor Horsley, however, has shown that the thyroid of the sheep most closely resembles that of man. M. Lannelongue said he derived his chief hope of its success from the way the engrafted thyroid in the case of a dog had not only grown well, but had prevented myxœdema. M. Chauveau thought it probable that the engrafted thyroid would be absorbed, and stated that it was an easy matter to engraft a sheep's testicle in subcutaneous tissue, but that the organ was gradually destroyed. Superficial capillary adhesions, he said, do not suffice to maintain the vitality of the transplanted organ; large vessels are needed for this purpose.

Another case of myxœdema has been reported, in which the operation of grafting the thyroid has been performed with success on a woman aged forty-one. M. Merklen showed the patient at a meeting of the Société Médicale des Hôpitaux in Paris. The graft, consisting of one of the lobes, was taken from a living sheep at the time of the operation, and was inserted beneath the skin in the right submammary region. No antiseptics were used, but the graft and wound were kept carefully aseptic, and healing occurred by first intention. The improvement appeared to be due in great part to the arrest of metrostaxis, from which the patient had previously suffered for months at a time. The hæmorrhage ceased three days after the operation, and had not recurred up to the time of reporting, some three months later. At this date the swelling of the face had decreased, the pseudo-lipomata diminished, and the speech become more natural.

Dr. G. Zuccaro has made some very careful enquiries upon the results of extirpation of the thyroid gland. These were especially conducted on dogs condemned to be slaughtered at the abattoirs. Dr. Zuccaro claims to have discovered that there exists in dogs, in addition to the thyroid gland, a lobule of different substance situated in front of the thyroid, and also two other accessory lobules of the same structure, but smaller at the posterior aspect of the gland. These, he says, correspond in structure to the noduli embrionali of Horsley, and the cumuli adenoidei of Virchow. Zuccaro found that when he removed the whole thyroid along with these lobules, the animals died, but when he left the lobules, removing the thyroid itself, that the animals survived the operation. After this he proceeded to remove the remaining lobules, and the animals died under the symptoms of cachexia strumipriva.

Dr. Zuccaro gives it as the result of his vivisections that, though dogs may survive the extirpation of the thyroid, they do not survive that of the three lobules, even should a portion of the thyroid be left. In conclusion, Dr. Zuccaro observes that the implantation of the thyroid, whether in the abdominal cavity, or in the subcutaneous tissues, is absolutely incapable of paralysing the effects of thyroidectomy, to which the animals succumbed, in the same way as if no preventive implantation had been effected. The reason of this is that the thyroid gland, after being fixed in its new destination, quickly becomes degenerated, and is absorbed.

Refs.: *Le Progrès Med.*, 1890; *Brit. Med. Journ.*, p. 1,258, ii., 1890; *Lond. Med. Rec.*, May 20, 1891.

